# Formation and radiative forcing of contrail cirrus

**DOI:** 10.1038/s41467-018-04068-0

**Published:** 2018-05-08

**Authors:** Bernd Kärcher

**Affiliations:** Institut für Physik der Atmosphäre (IPA), Deutsches Zentrum für Luft- und Raumfahrt (DLR Oberpfaffenhofen), 82234 Wessling, Germany

## Abstract

Aircraft-produced contrail cirrus clouds contribute to anthropogenic climate change. Observational data sets and modelling approaches have become available that clarify formation pathways close to the source aircraft and lead to estimates of the global distribution of their microphysical and optical properties. While contrail cirrus enhance the impact of natural clouds on climate, uncertainties remain regarding their properties and lifecycle. Progress in representing aircraft emissions, contrail cirrus and natural cirrus in global climate models together with tighter constraints on the sensitivity of the climate system will help judge efficiencies of and trade-offs between mitigation options.

## Abstract (web summary)

Condensation trails produced by jet aircraft contribute to anthropogenic climate change, yet the magnitude of their impact remains unclear. Here, the author reviews progress in understanding contrails since the last IPCC report (1999), assesses the remaining uncertainty, and identifies potential mitigating options and pathways forward.

## Introduction

Condensation trails (contrails) are line-shaped ice clouds generated by jet aircraft cruising in the upper troposphere at 8–13 km altitude. Depending on surrounding atmospheric conditions, contrails can be short- or long-lived. Long-lived contrails are those that remain for at least 10 min—defined by the World Meteorological Organization as Cirrus homogenitus^1^—and are the only man-made type of ice clouds. Depending on whether or not they retain their linear shape, they have been referred to as persistent contrails and contrail cirrus, respectively, or together as aircraft-induced clouds (AIC). A change in global cloudiness due to AIC creates an imbalance between incident radiation from the Sun and upwelling radiation from the Earth’s surface and atmosphere, resulting in a radiative forcing (RF) of climate that induces a tendency to change the temperature structure in the lower atmosphere.

Widespread recognition of a possible large-scale climatic influence of contrails dates back to several decades^2,3^. Prior to 1990, quantitative interpretation was hampered by a lack of knowledge of the properties of contrail cirrus, processes involved in their formation and tools and technologies to observe and represent them in models. A special report by the Intergovernmental Panel on Climate Change (IPCC) summarised and assessed in 1999 the scientific progress made regarding the climate impact of aviation^4^. While studies had begun to explore extensively the physical characteristics of persistent contrails, observational data on contrail cirrus were, and still are, very scarce, since it is difficult to distinguish them visually from naturally occurring high ice clouds (cirrus) owing to their irregular shape^5^, especially if their history is not known. Therefore, the IPCC special report did not provide a best central estimate of contrail cirrus RF. The resulting scientific uncertainty limited projections of the climate impact of aviation as a whole and prevented the formulation of options for mitigation. Since that report, climate model simulations have shown that RF due to contrail cirrus greatly exceeds that from persistent contrails^6^, making contrail cirrus the most important contribution to AIC and to RF associated with aviation. However, knowledge gaps still exist regarding the transition of contrails into contrail cirrus, optical properties determining their radiative response and the sensitivity of the climate system.

**Fig. 1:**
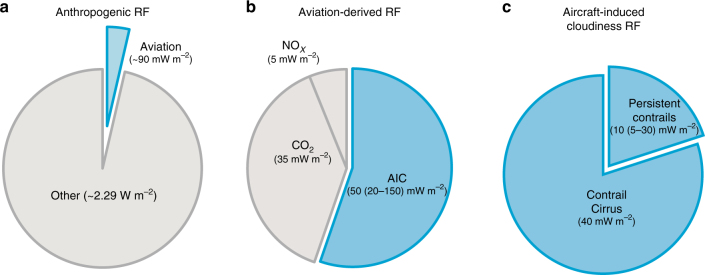
Aviation radiative forcing components. **a** Aviation as a percentage of total global radiative forcing (RF) due to human activities in the year 2011 relative to pre-industrial times, 2.29 (1.13–3.33) W m^−2^ (ref. ^7^). **b** Forcing components within the aviation fraction, of which aircraft-induced clouds (AIC) account for more than half. AIC and carbon dioxide (CO_2_) estimates represent 2011 emission levels^10^; the latter contribution has been obtained by extrapolating the year 2005 value^11^ using the corresponding increase in scheduled air traffic distance (Table 7.SM.2 in ref. ^10^). RF estimates for all aviation components together for the same year and emission levels superseding 2005 values are not available^13^. Aircraft emissions of nitrogen oxides (NO_*x*_) cause positive and negative RF contributions, values here calculated based on refs. ^107,139^. Small direct RF contributions from aircraft water vapour^140^ and particle emissions^14^ together nearly cancel out and are therefore not shown. No scientific consensus has been reached regarding the sign of RF due to modification of natural clouds by particle emissions. Therefore, the RF caused by those ‘indirect’ effects is not considered here, but may be of opposite sign and as large as that of AIC. **c** Breakdown of AIC radiative forcing into contrail cirrus and persistent contrails based on ref.^10^

The potential magnitude of the problem has given impetus to the need for improved understanding. The majority of RF driving climate change is due to anthropogenic emissions of warming (greenhouse) gases, most prominently carbon dioxide (CO_2_), and aerosol particles. In addition to CO_2_ emissions, civil aviation affects the climate through a number of non-CO_2_ climate forcing agents that are unique to this sector of transportation. Taken together, in the year 2011, these agents caused 4% of the total global RF from all human activities (Fig. 1). AIC represent the largest aviation RF component—comparable to RF induced by natural variations of the energy input from the Sun^7^—followed by aviation CO_2_ emissions and within AIC, contrail cirrus account for 80% of the RF.

Despite significant improvements in aviation technology and operation efficiency^8,9^, the steady increase in air travel volume—about 50% in terms of scheduled air traffic distance from 2000 to 2011 (ref. ^10^)—has led to concerns about the growing impact of aviation on climate. Depending on the economic projections and aircraft emission scenarios, aviation fuel usage and therefore CO_2_ emissions may double by 2050 (ref. ^11^). Some scenarios predict 3–4-fold increases^12^. When scaled to fuel usage, AIC RF relative to that of aviation CO_2_ emissions stays constant. However, such a scaling disregards non-linearity in atmospheric response to cloud changes. Exactly how this contribution will evolve also depends on changes in regional air traffic patterns and climate^10,11,13^. To eliminate AIC from the aviation climate equation, the research community needs to address knowledge gaps by advancing the scientific understanding of contrail cirrus and providing an assessment of options to mitigate the climate impact of AIC^14^. If the objective is to minimise the aviation contribution to climate change on long time scales (relating to centuries), then reductions in CO_2_ emissions are much more important than mitigating non-CO_2_ effects. However, persistent contrails and contrail cirrus have much shorter life times than CO_2_ emissions (hours versus centuries) and are therefore amenable to rapid mitigation.

This review brings together and draws upon knowledge on understanding their lifecycle and effects on Earth’s radiation budget since IPCC 1999. Determining conditions with predominant meteorological or microphysical control of contrail cirrus, connecting their radiative effects with global temperature change and exploring mitigation solutions will help develop appropriate technological and operational responses to reduce the climate impact of aviation as a whole and inform decision-making.

## Stages of contrail evolution

Natural cirrus, persistent contrails and contrail cirrus are high level clouds composed of ice crystals that form and evolve in ice supersaturated regions^15^. Ice supersaturation denotes sufficiently cold and moist atmospheric conditions—relative to saturation with respect to the ice phase—that influence radiative properties, extent and lifetimes of all cirrus cloud types and is required for contrails to be long-lived and evolve into contrail cirrus (Table 1). Duration and extent of ice supersaturated regions and the magnitude of ice supersaturation within them strongly depend on the synoptic (meteorological) situation and set upper limits for the lifetime and extent of AIC. Outside ice supersaturated areas in drier or warmer (ice subsaturated) air, contrails may still form, but are only short-lived and thus stay narrow with small areal coverage, leaving little to no impact on RF. Cirrus ice crystals form around microscopic aerosol particles. What sets AIC primarily apart from natural cirrus is the aircraft-dominated formation stage, wherein jet engine emissions, modified in aircraft wakes, result in a larger number of smaller ice crystals^5,16,17^ and therefore different evolution pathways and radiative effects. Understanding the processes at work in the formation and subsequent atmospheric spreading stage is key in determining the relative importance of aircraft/fuel-related meteorological and microphysical controls on AIC RF.

**Table 1:** Characteristics of contrails and contrail cirrus

AIC	Short-lived	Long-lived	
Ice cloud type	Contrail	Persistent contrail	Contrail cirrus
Morphology	Line shaped	Line shaped	Irregularly shaped
Meteorological condition	Ice subsaturated	Ice supersaturated	
Duration	0.1–10 min	10 min–10 h	
Depth	100 m	100–1000 m	
Width	10–100 m	0.1–10 km	<100 km
Length	0.1–10 km	0.1–10 km	<100 km
RF potential	Negligible	Small	Large

AIC subsume persistent contrails and contrail cirrus and potentially clouds either formed or modified by aircraft soot and sulphate particle emissions. In this review, the latter are not included in AIC due to lack of observational evidence of their existence. Contrails are line-shaped at formation and may attain irregular shapes due to non-uniform winds, turbulent (random) motions and humidity fluctuations at some point during their lifetime. Long-lived contrails spread out due to wind shear that is common at cruise altitudes. Those maintaining a linear shape are referred to as persistent contrails. Irregular-shaped contrail cirrus cannot be easily distinguished from natural cirrus hampering their observation. Dimensions of contrail cirrus clouds depend on poorly known morphologies and have not been studied systematically, but are likely highly variable. At northern middle latitudes, individual ice supersaturated regions may have lifetimes of several days^143^ and pathlengths exceeding 1000 km (ref. ^144^), but such cases are rare and mean values are significantly lower. Ice supersaturated layer depths ranging between 300 and 1300 m have been estimated from corrected radiosonde soundings^145–147^, often including many thin (few 100 m) layers

### Formation stage

The formation stage unfolds behind engines of cruising aircraft and lasts for about 10 min (ref. ^18^). Through a combination of models, laboratory and in situ measurements, the last two decades have seen significant advances in identifying and understanding thermo-, fluid-dynamical and microphysical processes in the formation stage. Understanding these processes is essential to model and predict initial AIC properties.

**Fig. 2:**
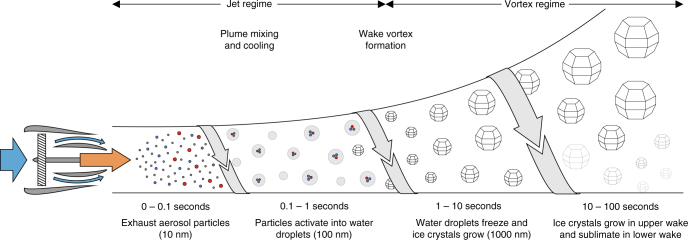
Processes influencing the contrail formation stage. Exhaust plumes produced by combusting fuel–air mixtures at high temperature and pressure within turbofan jet engines contain gaseous and particulate matter. Particles types present in the freely expanding and cooling plumes (jet regime) comprise emitted soot particles and aqueous aerosol particles formed in the exhaust (Box 1) along with entrained ambient aerosol particles. When turbulent mixing (arrows) and the associated cooling produces plume supersaturation over liquid water (*s*_w_ > 0), copious plume particles turn into water droplets (grey) that freeze and grow rapidly by uptake of water vapour forming a visible contrail. This happens when the ambient temperature, *T*, falls below the formation threshold, *Θ*. In soot-rich exhaust, most droplets contain soot inclusions. Droxtals, frozen water droplets with faceted surfaces, and hexagonal prisms and columns likely developing from them may provide a realistic representation of small ice crystals in fresh contrails, but no direct observational evidence for their shapes is available. Plumes from multiple aircraft engines merge with two wing tip vortices, forming an inhomogeneous wake. The further evolution of ice crystals depends largely on fluid-dynamical processes (vortex regime). The downward motion of the vortex pair warms the air causing ice crystal sublimation in the lower part of the wake. A small contrail fraction is released from the jets remaining at flight level and enhanced by detrainment of air from the lower wake. Ice crystals present in the upper wake continue to grow by uptake of entrained ice-supersaturated (*s*_i_ > 0) ambient water vapour. Few minutes past emission, the organised flow pattern collapses and mixes with ambient air (dissipation regime). Ice crystal images adapted from ref. ^141^ with permission from The Optical Society of America (OSA)

Contrails begin to form when jet engine exhaust plumes expand and their constituents mix with surrounding ambient air (Fig. 2). In line with aircraft observations^4^, a thermodynamic mixing model^19^ has shown that temperatures typically below 233 K (≈−40 °C) provide a threshold below which either short-lived or long-lived contrails appear behind jet aircraft cruising above ≈8 km (formation constraint). Contrail occurrence is predicted with confidence, if ambient pressure and relative humidity, water vapour and heat emissions, and propulsive characteristics of aircraft engines are known. The underlying definition of threshold temperatures rests on the assumption that exhaust plumes surpass water saturated conditions (water saturation constraint). The thermodynamic approach is not capable of predicting formation (nucleation) pathways and microphysical properties of ice crystals in contrails.

Aerosol particles types present in aircraft exhaust plumes comprise emitted soot particles, nanometre-sized (ultrafine) aqueous aerosol particles formed within the plumes and particles mixed (entrained) into the plumes from the ambient air (Box 1). In the cooling plumes, these particles interact with condensable vapours—mainly emitted water vapour—and ionised gas molecules. The different particle types compete for supersaturated vapour in water droplet formation; for current emissions, soot particles figure most prominently in droplet formation. Although water droplets are initially present in contrails, they are unstable since the upper troposphere stays below liquid water saturation^20,21^. Laboratory studies^22–24^ demonstrate that only after water droplets have formed does substantial ice nucleation occur, with rates of homogeneous (pure liquid) droplet freezing increasing dramatically with decreasing temperature (phase constraint). The presence of ice crystals in contrails is supported by ample observational evidence^25^. An analysis of aircraft observations shows that many (>10,000 cm^−3^) small (<1 µm) ice crystals need to form within a wingspan behind cruising aircraft to make contrails visible already at formation^26^ (visibility constraint). This is possible since plume cooling rates driven by turbulent mixing^27^ are high and plume particles acting as water condensation nuclei are abundant^28^.

Figure 2 indicates that properties of persistent contrails are further developed through circulatory airflow patterns (vortices) appearing behind aircraft wings. Plumes from multiple aircraft engines merge with the vortex pair, forming an inhomogeneous wake. The further evolution of ice crystals depends on the interaction between jet plumes and wake vortices. The vortex pair, in which most of the contrail is captured, descends some 100 m below the flight level. This downward motion leads to a partial loss of ice crystals due to sublimation in the lower part of the wake. Ice crystals present in the upper wake continue to grow by uptake of entrained ice supersaturated water vapour. After few minutes of contrail age, the wake-averaged ice crystal size distribution contains signatures of nucleation, growth and sublimation. Eventually, flow instability triggered by turbulence causes the organised flow pattern to collapse and mix with ambient air, terminating the formation stage. Evolution of ice crystals in engine plumes and aircraft wakes depend on ambient conditions and on the type of aircraft and jet engines, as borne out by fluid-dynamical simulations^27,29–33^ and remote sensing^34^ and aircraft^35,36^ measurements.

### Box 1 Ice forming particles in aircraft exhaust plumes

As mixing and associated cooling of jet plumes with surrounding air progresses, ambient aerosol particles are gradually mixed into them and exposed to moister and warmer plume air. Ultrafine aqueous particles (UAPs) are generated from gaseous emissions before ice crystals form. UAPs partition into a larger mode that formed on ionised molecules (chemi-ions)^41,128^ and an electrically neutral mode too small to contribute significantly to ice nucleation. Fuel combustion produces condensable vapours including water vapour, sulphuric acid, nitric acid, and low-volatile hydrocarbons. Sulphuric acid is produced by oxidation of emitted sulphur oxides and is highly water-soluble. Nitric acid is produced by oxidation of emitted nitrogen oxides and is only taken up by UAPs that are sufficiently diluted (water rich)^129^. The chemical nature of organic compounds from emissions of unburned hydrocarbons in aircraft exhaust is poorly characterised. The number of UAPs in the chemi-ion mode, exceeding 10^17^ per kg of fuel burnt^41^, is insensitive to variations of, and UAP sizes (1–10 nm) increase with, the sulphur content in the fuel^130,131^.

Incomplete combustion of carbonaceous phases present in aviation fuel produces aromatic compounds that merge to form elementary spherules. These solid black carbon (soot) aerosol particles grow further into fractal clusters mostly in the size range 10–100 nm and with numbers 10^14^–10^15^ per kg-fuel^93^. The fractal morphology of soot particles affects the relationship between total soot particle mass and number emissions. Their number and chemical state as well as the size gap between soot and UAP modes vary depending on engine type, engine power setting and type of fuel^93,132^.

Aerosol particles attract water molecules depending on their solubility and on relative humidity. Hygroscopic properties of liquid and mixed (liquid–solid) particles are determined by the chemical nature of the solutes. Insufficient knowledge of the size distribution and the surface-to-vapour interfacial energy (surface tension) renders model predictions of UAP activation into nearly pure water droplets uncertain. Hygroscopic properties of soot particles are mainly determined by chemical oxidants, condensation and particle scavenging processes in the jet exhaust plume producing thin aqueous coatings affecting their droplet formation (water activation) behaviour. In liquid water-supersaturated conditions, the largest and most soluble aerosol particles preferably activate into water droplets. Below contrail formation threshold temperatures, vigorous plume cooling rates (>10 K s^−1^) generate high supersaturation capable of activating the rather insoluble soot particles^28^. Modern jet engines extract more heat for propulsion^133^, meaning that plumes stay cooler and therefore develop higher supersaturation for similar water vapour emissions. Activation of plume particles ceases when the newly formed droplets start to quench the supersaturation due to condensational growth. This happens along with homogeneous water droplet freezing at plume temperatures 229–232 K (ref. ^28^).

The first particles to freeze are the water droplets originating from the largest soot particles and the more water soluble but much less abundant (equivalent to 10^12^–10^13^ (kg-fuel)^−1^) ambient particles. UAPs contribute to the pool of water droplets only if plume supersaturation is high enough to overcome the size (Kelvin) barrier for activation, i.e., if soot particles are removed from the exhaust to an extent that they no longer act as the primary condensational sink for water vapour and if temperatures fall significantly below formation threshold temperatures^37^. Desulphurising jet fuel decreases the size of UAPs and turns them into less potent droplet forming agents.

Laboratory studies suggest that exhaust water vapour by itself will neither form water droplets nor ice crystals^22^. About one third of aviation soot particles achieved a surface coverage of significant amounts of soluble material and activated into water droplets; ice formation set in at a relative humidity only slightly below liquid water saturation, where liquid water starts to nucleate ice homogeneously in UAPs^23^. However, the frozen soot fraction was very small (<5%), consistent with small amounts (1% by volume) of soluble coatings as found in other measurements^24^. Together, these findings support the notion of homogeneous droplet freezing as the predominant ice nucleation mode in fresh contrails.

### Simulating ice nucleation and sublimation

Robust predictions of changes in climate that result from AIC rely to a large degree on the ability to transfer the process understanding outlined above into models that are not capable of treating the formation stage explicitly due to a lack of spatial or temporal resolution. This goal can be reached by allying findings from observations with local scale, process-oriented models. While first attempts have been made to devise simulations addressing formation stage processes prior to IPCC 1999 (ref. ^4^), refined models have become available only in recent years^29–32,37–39^.

Together with observations, process models point to exhaust soot particles as the most important source of contrail ice crystals for current propulsion technology^5,37–40^. The ice nucleation process in contrails has been simulated assuming that the kinetics of water droplet and ice crystal formation are not affected by turbulent plume mixing and in which supersaturation is not resolved at scales (≈0.1–1 cm) below which molecular diffusion removes spatial gradients of water vapour and temperature. Water droplet formation followed by homogeneous freezing defines a minimal microphysical framework. By invoking a heterogeneous (solid surface assisted) ice nucleation mode involving bare exhaust soot particles, it is possible to tune these nucleation rates to trigger rapid formation of copious ice crystals preventing water supersaturation from occurring. However, this violates the water saturation constraint.

**Fig. 3:**
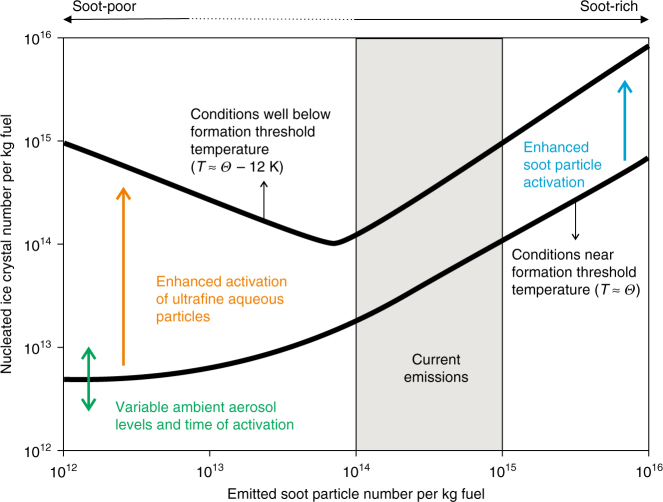
Nucleation of ice crystals in jet aircraft exhaust plumes. Ice crystal number emission index (per kilogram of fuel burnt) in the jet regime as a function of the number emission index of emitted soot particles simulated by a parcel model^37^. Two results are shown for an ambient temperature, *T*, close to a contrail formation threshold temperature, *Θ* ≈ 225 K, frequently found in extratropical cruise conditions, and a rather low temperature 12 K below this value. At intermediate ambient temperatures, nucleated ice crystal numbers increase due to enhanced water activation of either soot or ultrafine aqueous plume particles (Box 1). An approximate range of current in-flight soot emission indices is indicated (hatched area). In soot-rich exhaust, the number of soot particles that can be water-activated and freeze increases with decreasing ambient temperature that in turn increases plume cooling rates and levels of plume supersaturation over liquid water. As *T* approaches *Θ* in soot-poor exhaust, ambient aerosol particles mixed into exhaust plumes are the only source of contrail ice crystals, as increasingly fewer plume particles can be activated owing to diminishing plume supersaturation; the number concentrations of ambient particles are much lower in contrails than current soot emission levels. The plume cooling rate determines the time of water activation of entrained ambient particles, hence, the number of ice crystals derived from them. In soot-poor exhaust, ultrafine aqueous plume particles are formed in large numbers in the fresh exhaust, if the fuel contains sufficient amounts of condensable vapours besides water vapour. Those small particles are predicted to contribute significantly to ice crystal formation at low ambient temperatures well below average values at cruise levels (*T* ≈ 218 K in the extratropics and *T* ≈ 228 K in the tropics). If the formation of ultrafine particles cannot be avoided, ice crystal numbers are lowest in an intermediate range of soot emissions, 10^13^–10^14^ (kg-fuel)^−1^

Figure 3 shows the dependence of nucleated contrail ice crystal numbers as a function of soot particle emissions studied systematically by one process model based on the minimal framework^37^ (Fig. 2). The trends shown are generic and basically consistent with other parcel models^38,39,41^. In soot-rich exhaust, ice crystal and soot particle numbers increase nearly in proportion. Near the contrail threshold, only few ice crystals form, since low plume supersaturation diminishes water activation of soot particles. Ice crystal numbers decrease about 100-fold from soot-rich to soot-poor conditions, approaching constant low values set by the number of particles in the contrail environment. Ice crystal numbers increase at low ambient temperatures in soot-poor exhaust due to water activation and subsequent freezing of abundant aqueous plume particles (Box 1). While one observational case study is consistent with those predictions^42^, uncertainties in aircraft measurements of ice supersaturation and ice crystal number concentrations in fresh contrails have prevented direct verification of these results^28^. Simulations of soot-poor contrails remain unconstrained by observations, since current soot emission levels are high.

Computational fluid dynamics simulations are capable of predicting contrail properties past ice crystal formation in greater detail within limited spatial domains. While it is computationally too expensive to treat ice nucleation explicitly in large-eddy simulation (LES) codes, they have been employed to study effects of aircraft wake development on ice crystals. In this regard, LES have provided valuable insights into systematic dependencies of ice crystal losses on aircraft-dependent and atmospheric variables.

**Fig. 4:**
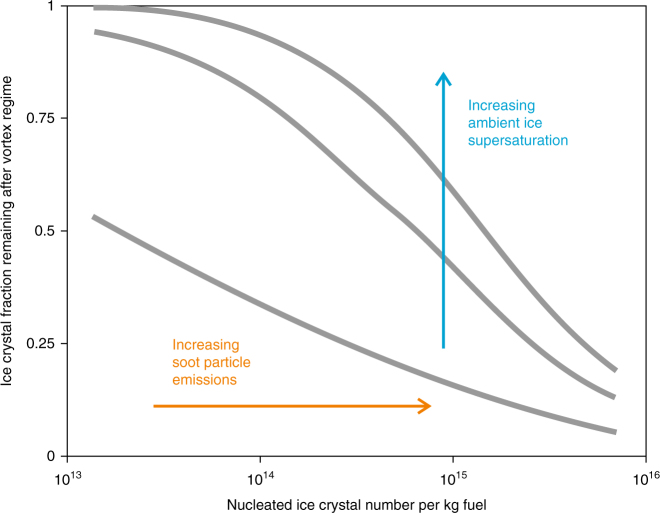
Sublimation-induced ice crystal losses in aircraft wakes. Dependence of the fraction of ice crystals escaping complete sublimation during the vortex regime versus nucleated ice crystal number emission index taken from LES simulations for a large aircraft at cruise and low (0.1), medium (0.2) and high (0.3) ambient ice supersaturation (Table A2 Block 4 in ref. ^43^). The magnitude of sublimation losses depends on aircraft engine thrust settings affecting soot particle numbers as well as on aircraft weight affecting the vertical depth of the wake; ambient conditions including ice supersaturation, air temperature and its vertical gradient and turbulence levels; and level of plume ice supersaturation and number, mean size and spread of the contrail ice crystal size distribution in the late jet regime. Substantial losses have been attributed to the Kelvin effect^31^, marking an increase in supersaturation over the surfaces of small particles relative to ambient values. Although this increase is very small (0.001–0.1) for µm-sized ice crystals, it becomes important in aircraft wake vortices for high ice crystal number concentrations causing rapid sublimation rates^42^

Figure 4 illustrates ice crystal sublimation losses as a function of assumed nucleated ice crystal number and their dependence on ambient ice supersaturation derived from one set of LES^43^, in which initial conditions of ice supersaturation and nucleated ice crystal properties in jet exhaust plumes have been prescribed. The more ice crystals nucleate, the larger the sublimation losses which decrease with increasing supersaturation. Observations confirm a reduction in ice crystal numbers in descending aircraft wakes^35,36,42^. First steps towards a unified description of these trends across different LES models have been taken^43^, but sublimation loss factors and ice crystal number concentrations remain uncertain until measurements become available providing tight links between soot emissions and ice crystal numbers across the entire formation stage.

Advances in research in the last two decades do not abrogate the need to measure more accurately physical and chemical properties of aerosol particles and ice crystals in the size range 1–1000 nm and to re-examine model predictions of ice crystal size distributions in and vertical extensions of contrails with better informed models at the end of the formation stage. These variables serve as initial conditions of AIC in the spreading stage and have important repercussions regarding the transition of contrails into contrail cirrus. It is important to ascertain model predictions by which sublimation decreases differences in nucleated ice crystal numbers induced by changes in soot emissions.

### Spreading stage

**Fig. 5:**
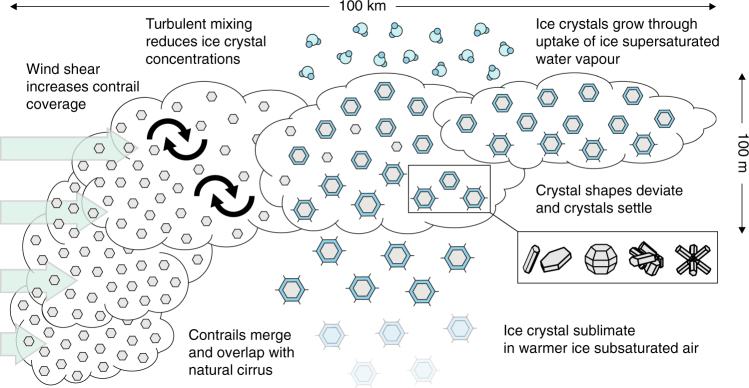
Factors affecting the development of aircraft-induced clouds. Areal cloud coverage increases due to vertical shear of horizontal wind components. Turbulent mixing (entrainment) forces ice crystal concentrations to decrease (dilute) over time. Ice crystal sizes increase by uptake (deposition) of water vapour from ice-supersaturated layers. Sustained deposition growth causes ice crystal shapes (habits) to deviate from initial isometry. The shapes of ice crystals, which are only poorly known and illustrated as droxtals, hexagonal prisms and columns, bullet rosettes and aggregates, affect their size-dependent growth and fall rates and optical properties. Ice crystals with maximum dimensions >30 µm settle (sediment) due to gravity with fall speeds >100 m h^−1^ and sublimate in warmer or drier air, while smaller crystals remain around the flight levels due to negligible fall speeds as long as some supersaturation can be maintained depending on the meteorological situation. The efficiency of sedimentation depends on the depositional growth rate, i.e., on ice supersaturation, the rate of cooling of air, and on ice crystal size, habit and number concentration. Sedimentation increases the vertical extent of AIC, which, in turn, enhances the rate of spreading and therefore coverage in a sheared flow. Sustained warming and drying due to large-scale subsidence dissolves AIC entirely. Ice crystal images adapted from ref. ^141^ with permission from The OSA

Microphysical and optical properties of ice crystals in contrails change as they spread or transition into contrail cirrus, depending on the meteorological situation and microphysical processes (Fig. 5). Over time, persistent contrails lose their initial linear shape and transition into contrail cirrus. They overlap and merge in traffic-congested areas, forming extended ice cloud layers that vary in shape, depth and lifetime and differ from natural cirrus in terms of microphysical, optical and geometrical properties. Ice supersaturated layers supporting them differ in vertical structure and horizontal area and the exchange of water molecules between vapour and ice phases within them. All these factors influence the RF potential of AIC (Box 2). Much of the knowledge about spreading contrails summarised below stems from space-borne observations^15,40^; little progress has been made since IPCC 1999 (ref. ^4^) in quantifying lifecycles of contrail cirrus and in identifying and understanding factors controlling them.

While formation conditions including those for short-lived contrails are frequently met at cruise altitudes^19^, the fraction of ice supersaturated areas in which aircraft actually fly is relatively small (10–15%) (ref. ^44^). Projected changes of extent and frequency of occurrence of ice supersaturated regions in the upper troposphere have implications for contrail formation^45^. Persistent contrails may merge with or form in natural cirrus^40,46–49^. AIC can be transported considerable distances (many 100 km) away from their source regions, therefore contrails and contrail cirrus may be found in areas where formation conditions are not met^50^. Over congested airspace, they manifest as ice cloud layers (‘contrail outbreaks’), which can extend over as much as 100,000 km^2^ and are readily apparent in satellite imagery^16^.

Optical properties of ice crystals influence AIC RF. Cloud optical depth (OD) measures the attenuation of radiation passing through cloud (Box 2) and is often reported at a wavelength in the visible part of the radiation spectrum. AIC RF is roughly proportional to OD and areal cloud coverage. Estimates of spatial coverage and radiative properties of optically thin (transparent) ice clouds depend on thresholds for determining their boundaries. Those thresholds could be a lower OD limit in remote sensing observations or a lower limit of ice water content in in situ measurements.

Satellite remote sensing reveals slight seasonal differences in contrail mean OD (average value 0.22) and particle effective diameter (36 µm) without diurnal variations^51^. Changes in the diurnal cycle of coverage and outgoing radiation in the North Atlantic region were estimated in correlation with air traffic^52^. Another study determined cloud properties formed during several contrail outbreaks that occurred over North America^53^. Both studies reported persistent contrails embedded in cirrus or sharing an atmospheric column with extant cirrus and suggested enhancements in OD and effective ice crystal size over the values found for contrails alone. A study quantified over the eastern North Pacific statistically the effect of contrails on OD of already existing cirrus, resulting in enhanced cirrus OD inside selected flight tracks, mostly in vertically thinner ice clouds^49^.

Some space borne observations include lidar measurements with increased temporal and spatial resolution of, and sensitivity to, optically thin contrails, assisted by manual detection^54^ or an automated tracking algorithm able to follow for some time a fraction of spreading contrails that are no longer line-shaped^55^. These studies have yielded values of mean geometrical thickness of 670 m, average contrail top altitude (temperature) of 10.9–11.7 km (≈219 K) and OD of 0.19–0.34, showing that contrails are mostly optically thin (OD < 0.3) in line with other studies^56^. Differences in the results of these studies reflect variability in microphysical and optical properties and meteorological conditions, differences in observation regions and air traffic activity, and inherent differences in detection methods.

Detectability of AIC from space depends on a large number of factors related to OD^15,57^. The optically thinnest ice clouds escape detection when using satellite-based, passive remote sensing methods that rely on upwelling radiance measurements, because detection efficiencies decline rapidly at low (<0.1) OD^58,59^. Meaningful comparisons of observed AIC properties, acquired via aircraft data sets^17,25^, with models are often precluded due to: missing information on contrail source aircraft and local formation conditions; sparse sampling of AIC that exhibit large spatial variability; poorly constrained dilution factors reducing ice crystal number concentrations; counting and chemically analysing small (sizes <0.01 µm) aerosol particles; counting and sizing errors in ice crystal size distributions taken with optical spectrometers; inability to directly measure with imaging probes the shapes of ice crystals with maximum dimensions <40 µm; and substantial uncertainties in measurements of supersaturation and ice water content—the amount of cloud ice per unit volume of air. Few attempts have been made^30^ to relate ice crystal size distributions of AIC from in situ measurements^17,60^ to microphysical simulations of the formation stage. While AIC might be detected in situ through a combination of measured aircraft co-emissions of NO_*x*_ and model-based trajectory analyses^17^, all types of observations remain inconclusive regarding the ability to separate contrail cirrus from natural cirrus due to the large variability in geometrical and microphysical properties of the latter. Moreover, it is difficult in dense traffic areas to determine whether natural cirrus have already been perturbed by aircraft emissions.

Space borne and aircraft measurements provide a fairly detailed picture of contrail properties, albeit with a considerable spread of observation results. While first steps resolving the transition of contrails into contrail cirrus have been taken, cloud system resolving models linking the regional scale evolution of AIC to specific meteorological situations and simulating their microphysical interactions with natural cirrus are lacking. To inform our incomplete understanding of the spreading stage, such models are needed along with collocated observations of mesoscale (1–100 km) vertical air motion and associated ice supersaturation variability as well as properties of ice nucleating ambient atmospheric particles^61^.

### Box 2 RF and climate sensitivity

At the top of the atmosphere, annually and globally averaged, incoming shortwave (SW, solar) radiation and the outgoing longwave (LW, thermal) radiation cancel out unless a climate forcing, such as changes in properties of clouds, perturbs this balance^134,135^. An initial perturbation of the radiation balance defines RF, a first-order indicator of the importance of different climate change mechanisms. RF refers to initial imbalances over a specified region and period of time; those provided by the IPCC^7^ are global averages taken relative to a pre-industrial time (1750). Global RF cannot be observed directly and is therefore obtained with the help of models or by extrapolation of regional values inferred from satellite observations.

OD—measuring the opacity of a medium when electromagnetic radiation of a given wavelength, *λ*, passes through it—is a crucial determinant of RF. OD and RF are determined by a number of radiative transfer processes. Ice in high level clouds, i.e., thin cirrus and long-lived contrails, acts similar to cloud-free air (**a**): it decreases the energy lost to outer space, enhancing the temperature in the atmosphere below the cloud and at the Earth’s surface (cloud greenhouse forcing). Cloud ice crystals affect RF by reducing thermal emission (radiant energy with *λ* > 4 µm) to space via absorption and re-emission of LW radiation at cold temperatures. Contrary to low clouds composed mainly of liquid water droplets (**b**), high ice clouds are often optically thin (**c**,** d**), i.e., partially transparent to solar radiation (*λ* ≈ 0.2–4 µm), meaning their ability to scatter SW radiation back to space (albedo forcing) is small. The greenhouse forcing is stronger for higher and colder clouds, in which ice crystals are increasingly abundant. The net average diurnal RF due to thin cirrus and contrails is a small positive residual from the wavelength-integrated SW and LW forcings. RF contributions due to thickening of natural cirrus by embedded contrails and new ice crystal formation in contrail areas with low ice crystal concentrations, blurring the distinction between natural cirrus and contrail cirrus, have not yet been determined.



Beside the power received from the Sun (solar irradiance) and Earth’s atmosphere, Earth surface conditions and vertical distribution (overlap) of clouds, wavelength dependent scattering and absorption properties of single ice crystals are needed to estimate OD and RF. Those properties depend on shapes (habits), surface roughness and orientation against incident light of cloud ice crystals across their size distribution, all of which are difficult to quantify in airborne measurements. Emissivity (absorption and emission at near infrared wavelengths, LW forcing) and reflectance (scattering at visible wavelengths, SW forcing) of high ice clouds are determined by OD and physical properties of ice crystals. Reflectance is sensitive to the habits of small ice crystals. Optical contrail models assume mixtures of various habits with fixed fractional weights^35,54,57,136,137^, but exactly which habits prevail and how individual habits vary with contrail age and ice crystal size is not well known.

Positive (negative) RF induces a warming (cooling) tendency that is subsequently communicated throughout the whole atmosphere. The strength of the resulting response in globally averaged, equilibrium near surface air temperature, Δ*T*, is estimated by the climate sensitivity parameter^134,138^, RF/Δ*T*. Values of this parameter and the associated equilibration times depend on the nature, spatial pattern and temporal evolution of RF and on the heat capacity of the climate system.

## Radiative and climate impact

Uncertainty in the understanding of the processes central to AIC formation and evolution is, to an extent, reflected in the tools used to estimate the associated RF (Box 2). Satellite observations can only provide OD and RF estimates for younger, line-shaped contrails, because these observations do not cover the full lifecycle of AIC^62^. Global climate models can augment such observations by providing OD and RF estimates associated with AIC of all ages including contrail cirrus to various degrees, depending on how they are represented in their cloud schemes. Crude estimates of changes in the global mean surface air temperature as a result of RF are only available for persistent contrails, not for contrail cirrus. Regarding changes of RF due to aircraft-induced modification of natural clouds, model studies have not led to robust estimates.

### Contrail variability

Variability in cloud properties—conveniently illustrated with the help of probability distribution functions (PDFs)—arises from variations in meteorological conditions and from cloud heterogeneity. Both sources of variability affect OD and hence RF and are not fully captured by global models. Large variability in microphysical and optical properties was observed in aircraft measurements of young (age <10–30 min) contrails^25,60,63,64^. In addition to satellite observations, high-resolution lidar^47^ and cloud models^65^ show marked variability in properties and RF of ageing persistent contrails.

**Fig. 6:**
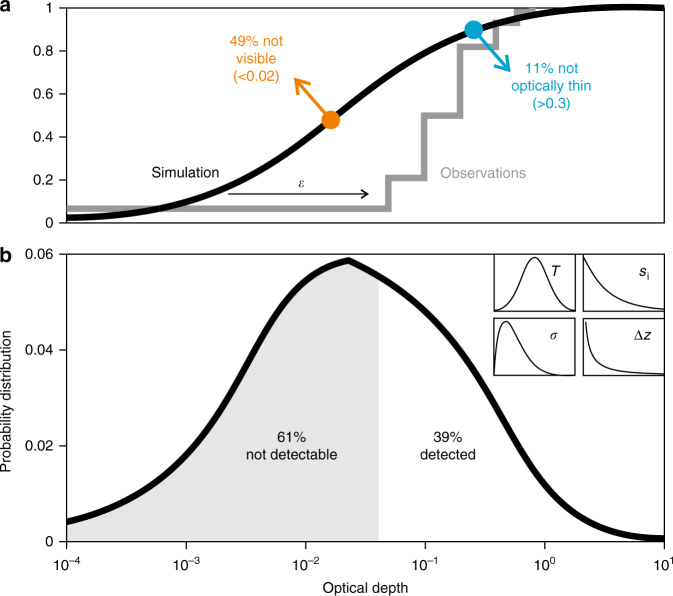
Variability in contrail optical depth. Probability distributions (PDF) of contrail optical depth, OD, and (top) the cumulative distribution (**a**) simulated by an idealised contrail model. The model PDF (**b**) was obtained (i) by solving an equation governing the evolution of the ice crystal size distribution accounting for mixing-induced dilution, shear-induced spreading, sedimentation and growth by vapour uptake and (ii) by imposing on this solution variability in meteorological factors described by probability distributions (inset) of air temperature, *T*, ice supersaturation (accounting for unresolved vertical air motion variability), *s*_i_, vertical shear of horizontal winds, *σ*, and thickness of ice supersaturated layers, Δ*z*, all constrained by atmospheric observations and numerical weather analyses. Simulation results^65^ are compared with satellite observations^56^ of contrails over the eastern North Pacific in the year 2001. According to the model analysis^66^, the measurements detected only 39% of all contrails over the whole OD range, 49% are subvisible (OD < 0.02) and 11% are not optically thin (OD > 0.3). By comparing simulated and observed PDF, detection efficiencies, *ε*, were inferred: 11.5% for data in the range 0< OD ≤ 0.05; 49% for 0.05 < OD ≤ 0.1; 94.5% for 0.1 < OD ≤ 0.2; and 100% for OD > 0.2, partly overlapping with values derived with other methods and in other conditions^59^. After adjusting the model PDF for inefficient detection using these efficiencies (horizontal arrow), it replicates the observed cumulant (stepped curve). Overall, applying the detection efficiency curve is consistent with a single cut-off OD value of 0.04, the upper limit OD value of the hatched area marking undetected (and partly invisible) contrails. The mean OD of all simulated contrails is 0.15, translating into 0.29 when corrected for non-detectable contrails. The corrected OD is roughly in line with the observed mean value, 0.24, that overestimates the model mean value of all contrails by ≈63%

Figure 6 displays schematically the characteristic shapes of variability of four key meteorological drivers of AIC development. Those PDFs drive a contrail microphysical model^65^ simulating in ice supersaturated conditions the dilution and spreading of contrails along with depositional growth and sedimentation of ice crystals within them. Figure 6 also depicts the resulting model PDF of contrail OD and its cumulant, along with an observed cumulative PDF^56^. The model PDF derives from an analysis of satellite observations^66^ and contains many optically thin and even invisible portions within contrails. This indicates that contrails that are non-detectable by passive remote sensing and invisible by ground-based observers may exert a non-negligible RF despite their low OD, since they are likely associated with large coverage^67^.

### Radiative forcing

**Fig. 7:**
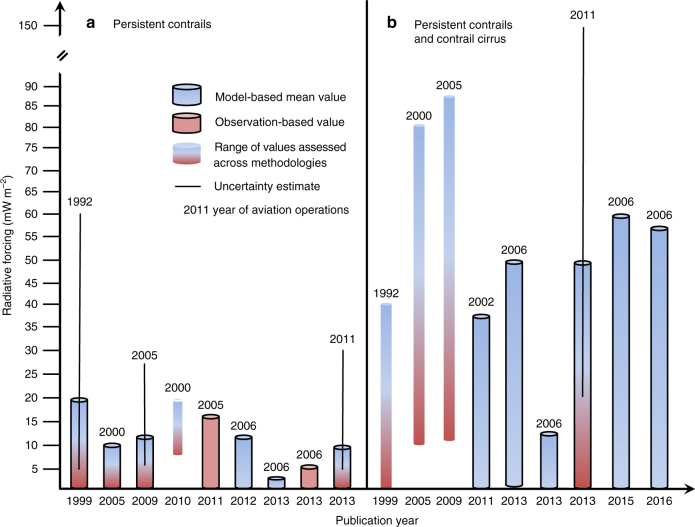
Global annual mean radiative forcing due to aircraft-induced clouds. RF due to **a** persistent contrails alone and **b** together with contrail cirrus from selected studies and assessments sorted by publication date since 1999. Purely observational global estimates derive from extrapolating local aircraft and regional satellite data. Pure model estimates are based on or rely upon global model simulations. Uncertainty ranges evaluated across several methodologies are taken from comprehensive assessments. IPCC 1999 assessed persistent contrail RF to be 20 (5–60) mW m^−2^ for air traffic in the year 1992 (ref. ^4^), followed by two studies summarising knowledge in the subsequent decade^11,73^. A narrower range, 8–20 mW m^−2^, was provided for the year 2000 after correcting older estimates that include off-line radiative transfer simulations with prescribed contrail coverage and mean optical depth for undetectable contrails^67^. Taken together, subsequent climate model simulations and extrapolation from in situ and satellite observations yielded 3–16 mW m^−2^ for air traffic in 2002–2006 (refs. ^63,69,71,142^). Relying in part on extrapolation and scaling data from earlier years, the IPCC most recently assessed contrail RF to be 10 (5–30) mW m^−2^ (ref. ^10^) for 2011. Refs. ^4,11,73^ provided possible RF ranges due to AIC. Ref. ^6^ estimated for the first time global AIC RF to be 38 mW m^−2^ for air traffic in the year 2002 using a climate model that tracks AIC explicitly; an improved model version^70^ estimated 56 mW m^−2^ for 2006. AIC RF values 50 mW m^−2^ and 60 mW m^−2^ were predicted for 2006 based on an off-line model that tracks contrail segments forming along flight tracks in combination with weather forecast and climate host models^52,88^. In a climate model that regards contrails as a source term for natural clouds, ref. ^69^ reported a global AIC RF of 13 mW m^−2^ for the same reference year. The IPCC best estimate of global net RF due to AIC for air traffic in the year 2011—associated with low confidence—is 50 mW m^−2^ (ref. ^10^), corresponding to more than half of the total aviation RF, with a 90% uncertainty range, 20–150 mW m^−2^. This most recent best estimate does not include indirect effects of aircraft particle emissions on natural clouds

Local radiative flux changes due to contrails can be as high as 100 W m^−2^ (refs. ^55,56^). In such rare cases their development may be affected by radiative heating^68^ enhancing vertical mixing. Regional contrail RF can be close to or above 1 W m^−2^ (refs. ^6,52,69–71^). Globally averaged, annual mean RF values and uncertainty ranges for persistent contrails alone (≈0.01 (0.005–0.03) W m^−2^) and together with contrail cirrus (≈0.05 (0.02–0.15) W m^−2^) are compiled in Fig. 7, with an emphasis on recent developments. Over time, early assessments of contrail RF have been basically confirmed and the large range of uncertainty has narrowed considerably.

Until 2011, a best central estimate of RF due to contrail cirrus including RF due to persistent contrails was not available. This caused large uncertainty in aviation RF as a whole. After IPCC 1999, AIC RF estimates have been based on associating air traffic with regional trends in cirrus coverage without proof of causality and by assuming equal radiative efficiencies of contrails and contrail cirrus^11,15,72,73^. The first RF estimate for persistent contrails and contrail cirrus was based on a global climate model that represents AIC as a separate cloud class^6,70,74^. An offsetting feedback of AIC on natural clouds due to humidity and (to a lesser degree) temperature changes was estimated with large uncertainty to reduce the direct AIC RF by almost 20% (ref. ^6^). The RF values compiled in Fig. 7 have not been corrected for this feedback. One climate model^69^ reports much lower AIC RF for 2006 aircraft operations than others, possibly because in the model contrails, while evolving consistently with the hydrological cycle, are undifferentiated from other clouds. The same model predicts an increase in AIC RF from 2006 to 2050 by a factor of seven due to a non-uniform regional increase in air traffic and different sensitivities for the forcing in different regions^75^. While model-based central estimates of AIC RF converged over time, results for current traffic levels are not available and the level of scientific uncertainty remains large.

### Surface air temperature changes

The climate sensitivity parameter serves as a measure of the RF-induced change in globally averaged, equilibrium surface temperature (Box 2). Climate sensitivities for persistent contrails have been derived from equilibrium climate change simulations with two different global models, yielding 0.43 K (W m^−2^)^−1^ (ref. ^76^) and 0.3 K (W m^−2^)^−1^ (ref. ^77^), meaning that contrails induce a smaller global temperature change per unit RF than CO_2_ emissions. In combination with the most recent IPCC uncertainty range in contrail RF of 5–30 mW m^−2^ (Fig. 7), these contrail climate sensitivities imply a global warming contribution of 0.00115—0.03 K. Both studies agree that this temperature response is only weakly correlated geographically with air traffic and dismiss previously suggested influences of contrails on decadal temperature trends over the U.S.A.^15,78^. Without specifying the AIC contribution, ref. ^79^ simulated for 2006 aviation operations a lower-limit global surface temperature increase of 0.01 K due to all aircraft climate forcings together.

The contrail climate sensitivities mentioned above are at the low end of the overall climate sensitivity uncertainty across climate models^7^, 0.4–1.2 K (W m^−2^)^−1^. Combining the latter range with the most recent (year 2011) uncertainty range for AIC RF, 20–150 mW m^−2^, leads to a highly uncertain equilibrium global warming contribution of 0.008–0.18 K due to AIC. This may be compared to the range 0.014–0.042 K due to accumulated aviation CO_2_ emissions alone inferred from the associated RF of 35 mW m^−2^ (Fig. 1), as well as to the estimate of 0.85 (0.65–1.06) K, the global temperature increase due to all anthropogenic activities accumulated over the period 1880 to 2012 (ref. ^80^).

More effort is required to understand the causes of spread in AIC RF estimates across different global climate models and methodologies. Total uncertainty estimates should account for correlations between uncertainties due to different processes. Besides a more complete representation (parameterisation) of AIC, an improvement of the representation of natural cirrus in global models^61^ is also needed to simulate AIC RF and its dependence on future air traffic patterns and climate change with greater confidence. Understanding forcing-response relationships in the climate system to better quantify surface air temperature changes warrants further scientific scrutiny^81^. In particular, time series of AIC RF and climate sensitivity estimates for contrail cirrus are needed to better constrain the contribution of AIC to global warming.

### Effects of aircraft particle emissions on natural clouds

Full quantification of aircraft-induced cloudiness changes is hampered by a lack of knowledge on whether and how particle emissions affect natural liquid phase and ice phase clouds. Climate models indicate that increases in upper tropospheric sulphate and soot particle mass concentrations due to aviation emissions are at least tenfold smaller compared to other sources^82^. They also suggest that aircraft emissions increase the number concentrations of fine (sizes < 100 nm) aerosol particles significantly in some areas^82,83^. Soot particles, regardless of their source, do not appear to contribute much to natural cirrus formation^84,85^. In contrast, sufficiently large sulphate particles tend to be efficient liquid cloud nucleating agents; several climate models predict significant modification of low level liquid phase clouds by emissions of small aircraft sulphate particles^82,83,86^. A global model study of the influence of soot particles emitted by aircraft and other sources on natural cirrus found RF estimates that would dominate the total aviation RF, but the sign of the simulated forcing could not be ascertained^87^. A contrail model run within a climate model predicted reductions in the total water column and high- and low-level cloud coverage^88^. Based on the assumption that aircraft soot particles—once preconditioned in contrails—act as highly efficient ice forming agents, one climate model predicted substantial effects on natural cirrus^89^. This relied, in part, on assumptions of how ice forms in background cirrus—a matter of current debate^61^.

Given the lack of observational evidence for effects of aircraft particle emissions on natural clouds and the poor model treatment of underlying processes, reducing scientific uncertainty and building confidence in these effects remain challenging.

## Mitigation

**Fig. 8:**
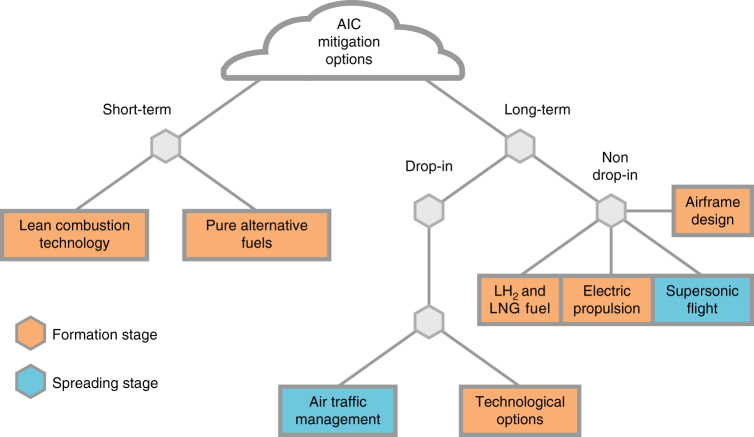
Options to mitigate the climate effect of aircraft-induced clouds. Options are categorised into short-term and long-term solutions. Long-term options are further distinguished as drop-in (easily replaceable) and non-drop-in approaches. Mitigation options may also be categorised according to their influence in the two stages of AIC evolution. Most options, including technological options addressing alterations of aircraft engine architecture and airframe/engine integration, affect the formation stage (red) by reducing or modifying particle emissions or affecting nucleation and sublimation of contrail ice crystals in aircraft exhaust plumes and wakes. Only two options affect the spreading stage (blue), requiring sound predictive capability of ice supersaturated areas (air traffic management, ATM) or producing short-lived contrails in the dry lower stratosphere (supersonic flight) with negligible radiative impact

The achievement of the 2015 Paris (COP21) agreement^90^ limiting global warming to 2 °C (preferably 1.5 °C) above pre-industrial levels by the end of this century, requires a dramatic pace for decarbonisation. If greenhouse gas emissions continue for just 15 more years, global surface temperature could rise as much as 3 °C (ref. ^91^). International aviation can help achieve the COP21 goals by mitigating the contribution of AIC to aviation RF on short time scales and thereby buy time to more substantially reduce aircraft CO_2_ emissions. Figure 8 summarises several short-term (relating to several years) and long-term (many decades) AIC mitigation options which are further discussed below. While some long-term solutions can be implemented gradually, others require changes in aircraft design, fuel production or supply infrastructure.

### Short-term solutions

Understanding how changes in fuel composition affect engine reliability and safety presents engineering and scientific challenges^8,9^. Replacement fuels are limited to those that pass both technical and safety requirements. Alternatives to kerosene from crude oil include synthetic fuels (produced by Fischer–Tropsch chemical conversion processes from coal, natural gas or biomass) and biofuels (from feedstock, algae or biomass)^92^. To meet technical and safety standards, those can be implemented rapidly only when blended with kerosene. Alternative fuels need to be assessed in terms of their environmental footprint including lifecycle analyses to ensure an overall carbon offset.

As pure synthetic fuels and biofuels contain next to no sulphur and aromatic species, their use would lead to a substantial reduction in ice forming aqueous plume and soot particles (Box 1). The use of kerosene-biofuel blends reduces soot particle emissions only moderately (≈−50%)^93^ and exerts a rather small impact on ice crystal numbers (−35%)^42^, since lower nucleated ice numbers due to reduced soot particle emissions (Fig. 3) are partly compensated by enhanced sublimation losses (Fig. 4). This means that they are unlikely to bring about a large reduction in AIC effects. The same is expected for kerosene-synthetic fuel blends. While a higher hydrogen content in kerosene-biofuel blends relative to conventional jet fuel (+8%)^93^ slightly increases contrail formation threshold temperatures^19^ (0.5–1 K), and thereby causes contrails to form more frequently, this does not lead to a significant increase in ice crystal numbers at the end of the formation stage (<5%)^42^. The effect of alternative fuels on contrail ice crystal formation is currently being investigated in airborne measurements^93^.

Pure biofuels cannot satisfy current supply demands and research into emissions arising from the use of pure synthetic fuels is still in the demonstration stage. It is therefore meaningful to explore the capability of modifications to propulsion technology to reduce in-flight soot emissions. Lean combustion technology (employing high air to fuel ratios during fuel combustion) produces low soot particle emissions by number in ground-based measurements (Table 1 in ref. ^94^). Other technical options to reduce contrail ice formation have been proposed^95^; their viability has not yet been demonstrated and they may become operative only in the long term.

The prospect of reducing in-flight soot particle numbers more than 10-fold using pure alternative fuels or lean combustion motivates climate mitigation studies of soot-poor contrail cirrus formed on low levels of ambient upper tropospheric aerosol particles (Fig. 3). The sulphur level in kerosene can be reduced without direct impact on engine performance, albeit with minor enhancements (≈0.1 %) in CO_2_ emissions^4^. Substantial reduction of the total number and mean size of exhaust soot particles along with desulphurisation of jet fuel is an efficient root level solution to curb AIC effects including potential indirect effects on natural clouds^96^. In the context of mitigation, this places investigations of climatic effects of aircraft particulate emission reductions right into the crosshairs of scientific scrutiny. To assess the associated benefits in an optimal way, aircraft emission inventories used in climate models should capture realistically the various sources of variability in soot particle emissions by number.

### Long-term solutions

Liquid hydrogen (LH2) and liquefied natural gas (LNG, consisting of mostly methane) have zero soot and sulphur emissions. In a climate model study, RF due to a hypothetical fleet of LH2-propelled aircraft (cryoplanes) with increased water vapour emissions was smaller than for kerosene aircraft^97^. Full electrification of future generations of aircraft would lead to zero emissions of CO_2_, NO_*x*_ and particles. In realising the vision of a transition to electric flight over the next decades, including transcontinental routes, a main technological hurdle is the power stored in batteries with sufficiently high energy density. Blended wing body technology combined with multi-fuel hybrid engines has been considered for civil aviation as a means to improve overall efficiency and reduce fuel consumption, hence, CO_2_ emissions^98^. A scenario assuming that one engine burns biofuel and the other LH2 (LNG) showed that AIC RF increases by 50% (40%) compared to conventional future technology due to changed contrail formation conditions^99^. While the effect of reduced soot emissions has not been investigated in detail, the study suggested that a reduction of AIC RF might be achieved when employing blended wing body technology together with soot-poor fuels.

The long-term transition to a fleet of supersonic aircraft operating in the lower stratosphere (altitudes 16–20 km) will increase levels of water vapour and nitric acid (deriving from aircraft NO_*x*_ emissions) due to longer lifetimes of emissions. The lower stratosphere is commonly too dry to support AIC, but those gaseous emissions affect polar stratospheric cloud formation with negative repercussions for the ozone layer^100,101^. Evaluating the climate impact of a hypothetical civil supersonic fleet without accounting for effects on stratospheric cloudiness, one study predicts a total RF of 22 (9–29) mW m^−2^ in 2050 (ref. ^102^).

Air traffic management (ATM) plays a key role in enabling safe and energy efficient operation. Studies have begun to explore the mitigation potential of changes in ATM with regard to wind-optimised improvement in fuel consumption^103^. The identification of those regions depends heavily on the accuracy of weather forecast and climate models. Re-routing strategies aim at reducing effects of non-CO_2_ forcing agents by flying in less climate-sensitive regions, while at the same time evaluating associated increases in economic costs^104^. Results based on simplified climate response models indicate that even small differences between various mitigation scenarios resulting from flight altitude variations might be reliably assessed^105^. One study suggests that circumnavigating the Arctic circle by re-routing cross-polar flights reduces soot pollution there^106^.

Trade-offs must be considered when applying technological and operational changes^8^. The chemical impact of contrail avoidance measures is considered small^107^. While entirely avoiding the formation of persistent contrails by re-routing is arguably difficult, it is desirable to identify and avoid meteorological conditions that cause the potentially largest climate impact: long lived, large scale, ice supersaturated areas^108^. Simply re-scheduling global air traffic to more daytime flights to promote the compensation of SW and LW AIC RF (Box 2), however, bears only a limited mitigation potential^109^, contrary to an earlier suggestion^110^. Other approaches study mitigation as a function of different weather situations^104^.

Most mitigation options affect the formation stage (Fig. 8). Adopting the two principal stages of AIC evolution discussed here as a conceptual framework in mitigation studies offers the possibility to systematically explore the implications for mitigation by analyses of subcomponents within each stage, i.e., the jet and vortex regimes within the formation stage^42^. Studies addressing the formation stage (Fig. 2) quantify changes in ice crystal number due to nucleation and sublimation. Studies addressing the spreading stage (Fig. 5) focus on changes in RF. Soot particle emissions and atmospheric ice supersaturation are key factors tying the two stages together.

The climate impacts resulting from RF due to AIC and CO_2_ emissions need to be placed on a common scale by the use of suitable metrics beyond RF. To compare the associated climate responses on various time horizons, climate change metrics other than RF are more suitable for AIC^111–113^. Choosing a time horizon depends on the specific climate policy the metric should serve and helps evaluate the relative importance of short-lived and long-lived climate perturbations.

## Future perspectives

### Indirect cloud effects of aircraft-induced aerosol particles

Robust RF estimates of this potential contribution to AIC RF are missing due to lack of observational evidence and uncertainties in underlying mechanisms and their parameterisation. Mechanisms include modification of ultrafine aqueous plume aerosols during transport from cruise altitudes to lower cloud levels and pre-activation of emitted soot particles in contrails. Given the small size and the substantial fraction of organic matter in aqueous plume particles and the largely insoluble nature of exhaust soot (Box 1), particles processed in ageing aircraft plumes^114^ are neither expected to be efficient liquid cloud-nucleating particles nor potent heterogeneous ice nuclei. This casts doubt on their capability to facilitate cloud water droplet or ice crystal formation in atmospheric conditions. Pre-activation refers to an increase in an aerosol particle’s freezing temperature after ice nucleation occurred. While pre-activation has been observed in laboratory settings, it is not known whether it occurs on aircraft-emitted, small (<100 nm) soot particles in the atmosphere or in contrails.

Liquid aerosol particles produced from gaseous aircraft engine emissions are influenced by fuel composition (Box 1) but are not well represented in emission inventories used to initialise global models. They must be specified to estimate the formation and evolution of ultrafine aqueous plume particles. The sulphur dioxide (SO_2_) emission index in kerosene varies widely around a mean value ≈1 g (kg-fuel)^−1^ depending on the origin of the crude oil^4^; most jet fuel has lower emissions. The fraction of SO_2_ converted to condensable sulphuric acid at emission (few %) and the mass and chemical nature of emissions of condensable organic vapours are not well known. With regard to aircraft-emitted soot particles, emission inventories only provide mass-based soot emissions^115–117^—often as a single, fleet-averaged surface-level value—from which particle numbers at cruise are inferred using empirical correlations. This seriously underestimates variability in soot particle numbers in contrails at formation.

To parameterise aqueous plume particle formation and contrail ice nucleation properly in climate models, it is necessary to inform global aircraft emission inventories with additional sulphate/organics mass and soot particle number emissions. Such improved inventories better represent aviation activity and allow for more realistic simulations of the formation stage, AIC RF and effects on natural clouds by way of parameterisation.

### Radiative characteristics of small ice crystals in AIC

Radiative transfer parameterisations commonly used in climate models are designed to represent effects of natural cirrus that are composed of ice crystals, whose mean maximum dimensions hardly fall below 10 µm. In dense flight corridors, AIC might on average be composed of such small ice crystals^17,118^. As neither geometric optics nor Mie theory accurately describe such cases, other optical models might be employed to provide more realistic parameterisations of ice crystal scattering properties in contrails and contrail cirrus. Shapes of small ice crystals are largely unconstrained by direct observations (Box 2), constituting a significant drawback in applying advanced optical parameterisations. Before being implemented in global models, such parameterisations might be tested in regional models providing more detailed information on ice crystal size and habit distributions. This better constrains SW radiative transfer calculations and helps improve estimates of AIC RF and climate sensitivity.

Solar radiation fluxes declined from the 1950s to 1980s and partially recovered since mid-1980s^119^. A statistically significant increase in broadband (wavelength-integrated) SW radiation at the Earth’s surface in cloudless scenes has been observed at several locations across the continental U.S.A. in the period 1996–2007 (ref. ^120^). The observed trend was almost entirely due to an increase in the diffuse irradiance, while the direct component remained virtually unchanged, ruling out a concomitant decrease in atmospheric aerosol OD as the sole cause of the increase. Recent analyses using diffuse SW spectral measurements^121^ confirmed this trend coined as ‘clear-sky whitening’. Ref. ^121^ hypothesised that while a decreased aerosol load did increase the total SW irradiance, an increase in subvisible contrail-generated ice crystal ‘haze’ repartitioned this increase from the direct into the diffuse component. Formation and spreading stage processes including radiative effects of small ice crystals must be understood well to explore this plausible hypothesis.

### Ice cloud parameterisations

On the one hand, instrumental challenges pose significant limitations in measurements of microphysical and optical properties of contrail cirrus. This heightens the role of models (Table 2) in providing RF estimates. On the other hand, improved scientific understanding is required before embarking on parameterisation.

**Table 2:** Challenges in modelling radiative forcing due to aircraft-induced clouds

Model scale	Spatial resolution		Contrail stages	Major challenges	Approach/solution
	Horizontal	Vertical			
Local	<10 m	<10 m	Formation stage	Ice crystal number and size distribution	Turbulence–microphysics coupling
Regional	<1000 m	<100 m	Spreading stage	Radiative flux changes and interaction with natural clouds	Contrail to contrail cirrus transition
Global	<100 km	<1 km	Full life cycle	Ice crystal formation and ice supersaturation	Parameterisation and high resolution

Improvements in a hierarchy of local- to global-scale models to be realised in conjunction with observations providing data for cloud and radiation parameterisation development and overall model validation

Local scale process and fluid-dynamical models systematically explore how plume turbulence and wake dynamics affects nucleation and properties of contrail ice crystals (Fig. 2) and how predictions of nucleated ice numbers (Fig. 3) and sublimation losses (Fig. 4) relate to aircraft measurements. Cloud-resolving and regional models—driven with appropriate meteorological boundary conditions and combined with optical parameterisations capturing the SW response of µm-sized ice crystals—analyse large atmospheric regions based on collocated aircraft and satellite measurements to better judge AIC RF and the radiative significance of natural cirrus perturbed by contrails. These models capture variability in ice supersaturation and are employed to drive LES to gain more detailed understanding of the contrail-to-contrail cirrus transition. Global models use information regarding properties of thin ice supersaturated layers (Fig. 5) derived from high-resolution simulations or radiosonde data^122^ to improve their representation of AIC supporting areas^50^. They also use parameterisations of formation stage processes to initialise AIC properties and rely on progress in simulating the formation of natural cirrus more realistically^61^.

Global models represent much of the system complexity, but account for processes and their variability affecting RF only to a degree, depending on the model’s representation of clouds. They track atmospheric motion and clouds with ≈10 min time steps and represent them with coarse spatial resolution. This means that formation and effects of clouds must be parameterised. Cloud microphysical and optical variables evolve on unresolved temporal and spatial scales. The connection of unresolved processes to resolved (large-scale) model variables is not always obvious. With higher resolution, fewer parameterisations are required. The prospect to perform in the near-term global studies that resolve explicitly the regional scales crucial for AIC development^123^ is very promising. More aircraft travelling in the same regions may not necessarily lead to more AIC coverage due to overlap effects and merging with natural cirrus, thus limiting increases in RF for higher air traffic volume. Moreover, AIC and natural cirrus do not evolve independently^6,49^. A new generation of high-resolution global models explores such effects in the spreading stage with increased confidence.

Improving and validating AIC in global models requires an integrative analysis framework that unravels crucial cloud-controlling factors and conditions. Propitious weather conditions leading to large radiative impact of contrail outbreaks are tied to synoptic-scale, enhanced moisture transport and uplift (cooling), in which moderately high ice supersaturation at the same time minimises sedimentation losses and maximises OD by generating ice crystals within an optimal size range^31^, reminiscent of the ‘Goldilocks principle’. A suitable framework combines aircraft and active remote sensing observations and local and regional models to study characteristics of and linkages between individual processes (bottom-up) with passive remote sensing observations, and global models to explore large-scale constraints arising from the coupled dynamics-aerosol-cloud-radiation system (top–down).

### Pathways to model improvement and data-model comparisons

Methodologies combining estimates of contrail coverage from meteorological reanalysis data and mean contrail OD in off-line radiative transfer models and used to diagnose global contrail RF in climate models have been replaced by simulations of AIC within global models^62^. Adding or elaborating on processes may be required to improve on specific problems. However, this would expand the space of underlying model parameters, possibly compromising the robustness of the models’ predictive capability and the goal of reducing overall model uncertainty^124^. Parameterisation schemes used in such models should therefore be informed by theory, whereby it is important to capture the most influential processes governing a specific application, and document the physical soundness and plausibility of the scheme. Understanding how well global climate models work for a specific purpose (evaluation) is important, but in itself is not a straightforward task. At times the disagreement of a model with observations motivates model improvements.

Another important task in support of model improvement is ascertaining its “truth” (validation), e.g., by comparison with suitable measurements. In models with many compensating uncertainties, several pathways of process representation conforming to the same set of underlying physical laws may lead to similar plausible agreement with observations. Just because models agree with some measurements does not imply reduced uncertainty in their RF predictions. In view of the case study nature of many measurements, observationalists and modellers should aspire to clarify if and exactly how measurements can be used to validate models and constrain uncertainty. Introducing microphysical mechanisms in cloud parameterisations without critical analysis, theoretical underpinning or support by empirical evidence increases the likelihood of model fallacies. Unexamined process representation carries the danger of proliferating unsubstantiated conclusions. The resulting enhanced uncertainty levels are difficult to judge and may be irreducible.

Lack of rigorous model evaluation and validation has serious repercussions for the interpretation of model results. A way out could be to devise models based on a minimal set of processes with only a few open physical parameters and expounding meaningful ways of how model behaviour could be confirmed or falsified. Complexity would be added only when needed, fulfilling a methodological principle known as ‘Ockham’s razor’. To minimise AIC model uncertainty, ice cloud parameterisations should conform to a number of tenets: full inclusion of AIC into the model’s hydrological cycle, as cloud-induced changes in the moisture and heat budget cause feedbacks on natural cloudiness; consideration of vertical overlap between AIC and natural clouds, as cloud overlap statistic has a profound impact on overall fractional cloud coverage within a model grid box and on net RF; realistic representation of AIC within the model’s radiation scheme, as optical properties differ from those of natural cirrus; and abandonment of instantaneous conversion of water vapour above ice saturation into cloud ice (and the reverse process below ice saturation), as ice crystal growth and sublimation can be kinetically hindered in thin or aged contrail cirrus; moreover, the kinetic treatment of water vapour uptake involving size-dispersed populations of disparate ice nuclei is a perquisite for the proper treatment of ice nucleation in models. Implementing these improvements will also lead to better simulations of natural cirrus.

### Reducing the impact of AIC on climate

Commercial aircraft are in service for 20–30 years meaning that adoption of new technologies from research to integration takes many decades. Therefore, in addition to scientific challenges, the implementation of AIC mitigation options also faces engineering and economical hurdles and will only be realised when disruptive change in the air transportation system can be avoided. Accomodating the expected growth in air travel while reducing the impact of AIC RF may require a combination of options.

The International Civil Aviation Organization (ICAO) agreed upon a carbon offsetting and reduction scheme for global aviation^125^, but does not explicitly address AIC in their environmental protection activities. Integrating AIC in these activities would constitute an important stepping stone in support of widespread implementation of AIC mitigation options. The use of biofuels has already been initiated by the aviation industry in the necessary transition away from fossil-based fuels. Besides implementing a standard for carbon neutral growth, ICAO has also developed certified testing procedures applying from 2020 for particulate emissions from aircraft engines based on particle mass and number^125^. This action facilitates the realisation of mitigation solutions addressing formation stage (emission-related) processes and holds promise to reduce AIC effects on climate on short time scales^96^. With regard to the spreading stage, while an interim strategy has been conceived^126^, ATM is considered to offer a potential long-term solution as meteorological forecast skills mature and if ATM proves to be flexible enough to allocate climate-compatible flight routes quickly and safely.

Compelling evidence has been presented as to the urgency to prevent human activities from causing unacceptable environmental change^127^. Studying climate change in response to AIC involves basic scientific challenges and is important to address in the larger context of how anthropogenic activities affect natural cirrus clouds and climate^61^. Better understanding the radiative response of contrail cirrus as it emerges from coupled meteorological and microphysical controls requires an integrative approach aiming at understanding on the process level at small scales and at the same time be cognisant of interactions that operate on large scales.
